# Type 2 diabetes across populations of different ancestry: deconstructing the genomic landscape

**DOI:** 10.1038/s41392-024-01944-8

**Published:** 2024-09-09

**Authors:** Georgia Xourafa, Christian Herder, Michael Roden

**Affiliations:** 1 Institute for Clinical Diabetology, German Diabetes Center, Leibniz Center for Diabetes Research at Heinrich-Heine-University Düsseldorf, Düsseldorf, Germany; 2 German Center for Diabetes Research, Partner Düsseldorf, München-Neuherberg, Germany; 3 Department of Endocrinology and Diabetology, Medical Faculty and University Hospital Düsseldorf, Heinrich-Heine-University Düsseldorf, Düsseldorf, Germany

**Keywords:** Endocrine system and metabolic diseases, Genetics research, Endocrine system and metabolic diseases, Genomics

In a recent study published in *Nature Medicine*, Smith and colleagues used data from 1.4 million individuals across 37 genome-wide association studies (GWAS) reflecting different genetic ancestral populations to identify 12 genetic clusters, mostly shared among the different populations, and associated the weighted sums of genetic variants of each cluster—termed as partitioned polygenic scores (pPSs)—with various lab-based, anthropometric and cardiometabolic traits. Their analyses extended previous studies on biological mechanisms linking genetics to type 2 diabetes (T2D) risk and pointed to both similarities of genetic clusters across multiple populations and differences regarding their contributions to overall T2D risk among different ethnicities.^1^

Over the last years, the substantial variation of the key abnormalities characterizing T2D: insulin resistance, and pancreatic islet dysfunction (resulting in relative insulin deficiency), among many cohorts has gained increasing interest and suggests divergent pathogenic mechanisms underlying the various metabolic disorders, presently summarized as T2D.^2^ Furthermore, phenotype-based clustering of T2D cohorts allowed identification of sub/endotypes with specific differences in the risk of diabetes-related comorbidities and complications.^2^ While heritability is a key feature of the etiology of T2D, individual genetic variants were previously reported to have only a very modest impact on the risk of diabetes and its comorbidities. This suggests that only robust meta-analyses of large GWAS might be able to detect distinct genetic clusters of diabetes and thereby help better understand the pathogenesis and improve targeted management of T2D and related complications.

**Fig. 1:**
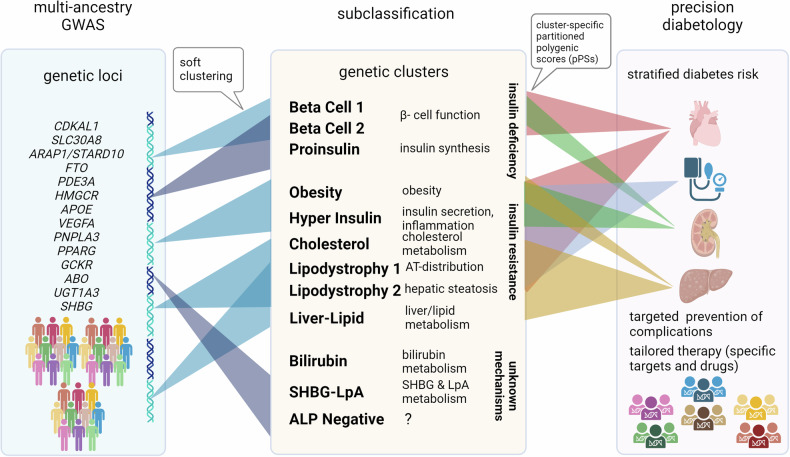
Identification of genotypic clusters by application of partitioned polygenic scores to multi-ancestry GWAS. Most relevant genetic variants enabling identification of specific clusters by soft clustering. These clusters associate with certain phenotypic and/or metabolic traits, which may allow for clinical translation by improving prediction of T2D and its associated complications after implementation of cluster-specific partitioned polygenic scores (pPSs)

The recently introduced concept to link pPSs with distinct clinical features and metabolic traits combines both genome- and phenome-based concepts of subtyping.^3^ Given the fact that the majority of people with diabetes worldwide belong to non-European populations, differences in genetic ancestry in attempts of subclassification cannot be ignored. Using a soft clustering method for the analysis of multiple ancestry-based data, Smith and colleagues identified 12 genetic clusters, of which three can be related to insulin deficiency (“Beta Cell 1”, “Beta Cell 2” and “Proinsulin)”, and seven to insulin resistance (“Obesity”, “Lipodystrophy 1”, “Lipodystrophy 2”, “Hyper Insulin”, “Cholesterol”, “Liver-Lipid” and “ALP Negative”), whereas two were related to currently unclear mechanisms (“Bilirubin” and “SHBG-LpA”) (Fig. 1). Compared to previous clustering studies based on GWAS data from European populations,^3^ the clusters associated with insulin deficiency were consistently recaptured, whereas those of insulin resistance differed.^1^ These findings might suggest a greater than expected role of genetic ancestry-related variation for insulin resistance, which might impact on tailored prevention and treatment of T2D and its complications. Both “Lipodystrophy 1” and “Lipodystrophy 2“ included *PPARG* among the top-weighted genes, which is known for its role in adipose tissue differentiation and as target of the insulin sensitizing thiazolidinediones. Interestingly, “Lipodystrophy 1” was indeed related to increased visceral adipose tissue volume, whereas “Lipodystrophy 2” was related to increased liver enzymes. Both pPSs were also linked to increased risk of metabolic dysfunction-associated steatotic liver disease (MASLD), which underlines the tight inter-tissue crosstalk between adipose tissue and liver during the development of T2D and MASLD.^4^ The multi-ancestry analyses of the correlation of BMI with T2D risk further identified these clusters as causal factors of the susceptibility to T2D at lower body mass index (BMI) in East Asian populations, implying their potential utility in stratifying BMI targets in the prevention of T2D. However, it appears that additional genetic mechanisms may be responsible for the lower BMI thresholds related to T2D risk in other non-European populations.

Of note, different genomic-based clustering methods do not uniformly yield congruent results. Another recent GWAS analysis of more than 2.5 million individuals, of whom about 40% were of non-European ancestry, including pPSs in a further 279,552 persons used a hard clustering method and revealed only eight non-overlapping clusters with one lipodystrophy cluster.^3^ Among the other clusters proposed by Smith and colleagues, some require special attention. The “Bilirubin” cluster exhibits a positive association between T2D risk and circulating bilirubin concentrations without connection to insulin resistance or deficiency (Fig. 1). This is neither supported by the other recent clustering analysis nor by a recent Mendelian randomization analysis, which showed a significant association between hyperbilirubinemia and decreased T2D risk.^4^

The “Cholesterol” cluster exhibited an association of the *HMGCR* locus, which encodes 3-hydroxy-3-methyl-glutaryl-coenzyme A (HMG-CoA) reductase, with lower LDL-cholesterol levels. This finding is intriguing as it suggests that some mechanisms may have opposite effects on the risk of T2D and coronary artery disease. The “SHBG-LpA” cluster results from merging of two formerly separate clusters. This calls for careful interpretation in the absence of other evidence for a common contribution of the two traits - lipoprotein A (LpA) and sex hormone-binding globulin (SHBG) to the development of T2D. Nonetheless, the new “SHBG-LpA” cluster confirms the previously suggested inverse causal association between SHBG and T2D risk.^4^ Despite the absence of an association of this cluster with indices of insulin resistance, the cluster-specific enrichment epigenomic annotations in fetal hepatoblasts may be related to the confirmed inverse association of SHBG with intrahepatic lipids. While this study attributes this association to males, such evidence has been reported before only for females.^5^

It is interesting that Smith and colleagues found differential associations of most of the twelve clusters with hypertension, coronary artery disease and MASLD.^1^ However, the statistical power to reveal cluster-specific associations with ischemic stroke, diabetes-related retinopathy and neuropathy was rather low suggesting the need for even larger cohort studies and for including analyses of diabetes-related cancers. These uncertainties as well as the restricted access to the still expensive GWAS analyses limit the broad worldwide application in the clinical practice of diabetes management in the near future.

In conclusion, pPSs derived from multi-ancestry GWAS data made it possible to propose a novel gene-based subclassification to improve the description of the disease complex, still termed type 2 diabetes mellitus. Although the novel genetic clusters were largely reproducible across different ethnicities, the ancestral differences observed among the different attempts of diabetes subtyping call for further large-scale studies with greater diversity.
